# Novel Technique to Reconstruct Peri-Implant Keratinised Mucosa Width Using Xenogeneic Dermal Matrix. Clinical Case Series

**DOI:** 10.3390/dj12030043

**Published:** 2024-02-20

**Authors:** Attila Horváth, Péter Windisch, Dániel Palkovics, Xinda Li

**Affiliations:** 1 Department of Periodontology, Semmelweis University, 1088 Budapest, Hungary; 2 Evident Pro Private Practice, 1118 Budapest, Hungary

**Keywords:** porcine dermal matrix, dental implant, peri-implant keratinised mucosa, attached mucosa, peri-implantitis

## Abstract

Reconstruction of sufficient buccal peri-implant keratinised mucosa width (PIKM-W) is reported to reduce the symptoms of peri-implantitis. In order to reduce the drawbacks of autogenous graft harvesting, we investigated a novel porcine dermal matrix (XDM, mucoderm^®^) using a modified surgical technique for augmentation of PIKM-W. Twenty-four patients were recruited with insufficient (<2 mm) PIKM-W. After split thickness flap preparation, the XDM was trimmed, rehydrated and tightly attached to the recipient periosteal bed using modified internal/external horizontal periosteal mattress sutures via secondary wound healing. Change of the PIKM-W and dimension of the graft remodelling were evaluated at 6 and 12 months postoperatively. The mean PIKM-W changed from 0.42 ± 0.47 to 3.17 ± 1.21 mm at 6 M and to 2.36 ± 1.34 mm at 12 M in the maxilla and from 0.29 ± 0.45 mm to 1.58 ± 1.44 mm at 6 M and to 1.08 ± 1.07 mm at 12 M in the mandible. Graft dimensions decreased by 67.7 ± 11.8% and 81.6 ± 16.6% at 6 M, and continued to 75.9 ± 13.9% and 87.4 ± 12.3% at 12 M, in the maxilla and mandible, respectively. Clinical parameters showed statistically significant intra- and intergroup differences between the baseline and 6 and 12 months (*p* < 0.05). The present technique using the XDM was safe and successfully reconstructed PIKM-W in both arches. The XDM alone seems to be a suitable alternative to autograft for PIKM-W augmentation in the maxilla.

## 1. Introduction and Background

The increased use of dental implants in replacing missing teeth is today associated with several challenges. The most common concerns are peri-implant mucositis (prevalence between 19 and 65%) and peri-implantitis (frequency in the range of 1–47%) [1,2,3,4,5]. The aetiology behind peri-implantitis seems to be multifactorial. The most common causative factors are considered to be poor plaque control, history of periodontitis, smoking, diabetes, lack of maintenance visits, lack of sufficient bone volume, iatrogenic factors (e.g., improper patient/implant selection, suboptimal surgical procedure, malpositioning, overheating, over-torque, overloading, excess luting cement), and, last but not least, lack of sufficient vestibulum depth and width of the attached keratinised mucosa [5,6,7]. Wennström (1994) showed that there were no significant differences in the maintenance of peri-implant health in sites with and without an adequate width of keratinised mucosa [8]. However, later, Romanos and co-workers indicated that the presence of an adequate width of PIKM might result in less plaque accumulation, less recession, improved soft and hard tissue stability and a lower incidence of peri-implant mucositis [9]. Roccuzzo et al. demonstrated that when the buccal peri-implant keratinised mucosa width (PIKM-W) was less than 2 mm, the implants were more prone to plaque accumulation as well as gingival recession, even in patients exercising sufficient oral hygiene and undergoing professional periodontal therapy [10]. Furthermore, in the absence of PIKM, increased plaque score, recession and bone loss could be detected [11,12]. In a recent study conducted by Gharpure et al., it was shown that implants with PIKM-W measuring less than 2 mm exhibited a significantly greater prevalence of peri-implantitis (prevalence ratio [PR] = 1.87), peri-implant mucositis (PR = 1.53) and pain or discomfort experienced while tooth brushing (PR = 2.37) compared to implants with appropriate PIKM-W [11]. Previous studies have shown that a decrease in PIKM-W is linked to a development in the formation of biofilm, inflammation of the soft tissue, heightened patient discomfort, recession of the mucosal tissue, loss of marginal bone and a higher occurrence of peri-implantitis [13]. Moreover, it was determined that there exists a favourable correlation between a PIKM-W greater than 2 mm and the general condition of the peri-implant complex [14].

In order to overcome the above complications, surgical enhancement of PIKM was suggested with autogenous or xenogeneic soft-tissue grafts. Previous papers have provided insufficient reliable evidence to provide recommendations on whether the techniques to broaden the PIKM or attached mucosa are beneficial to patients or not [15]. However, the recent literature has indicated that soft-tissue grafting treatments have been associated with improved peri-implant health outcomes. (i) In the reconstruction of the keratinised mucosa, autogenous grafts have been found to be effective in improving bleeding indices and promoting higher levels of marginal bone. (ii) Autogenous grafts have been shown to be a suitable method for increasing the mucosal thickness, resulting in much-reduced marginal bone loss [1]. Finally, free autogenous gingival grafts are considered the surgical standard to increase PIKM-W effectively. However, xenografts may be successful alternatives, since similar results were reported compared to autografts [13].

Free gingival graft, or, more accurately described, epithelialised connective tissue graft (ECTG), represents the gold standard as it results in minimal postoperative soft-tissue contraction and maximal predictability [16,17,18,19,20,21]. However, harvesting the ECTG may cause significant pain and discomfort at the donor sites (hard palate, maxillary tuberosity). Furthermore, the available amount of tissue is limited, and a colour match at the recipient site might be compromised [17,18,22,23].

To overcome the limitations of ECTG, authors have suggested using a xenogeneic collagen matrix (XCM) to enhance the PIKM-W. The existing literature shows that the XCM results in satisfactory outcomes in increasing the width of keratinised mucosa. However, the XCM undergoes significant contraction (resorption) due to its porous surface, resulting in far lower PIKM-W during secondary wound healing than is the case with ECTG [24,25]. As an alternative, a recently developed xenogeneic dermal matrix (XDM, mucoderm^®^) of porcine origin can be utilised, as this material undergoes less contraction, presents a quick rehydration time and slow resorption, facilitates angiogenesis and attracts fibroblast and osteoblast [26,27,28]. In order to test the potential effective graft materials for increasing PIKM-W, we conducted a pilot study including ECTG, sub-epithelial connective tissue graft (SCTG), allogenic dermal matrix (ADM), XCM and XDM. Six months postoperatively, the ECTG (3.5 ± 1.5 mm) and XDM (3.0 ± 1.6 mm) had created >2 mm PIKM-W compared to the SCTG (1.0 ± 0.0 mm), ADM (2.1 ± 1.0 mm) and XCM (1.5 ± 2.1 mm), respectively [29].

Therefore, the present prospective case series aimed to evaluate the efficacy of the novel XDM to widen the band of the buccal keratinised mucosa at 6- and 12-month observation periods by comparing the upper and lower jaws. In addition, we aimed to provide a detailed description of the surgical procedures we developed. According to the best of our knowledge, no study has investigated the above parameters.

**Null Hypothesis 1:**  *The use of this XDM with a modified surgical technique does not significantly widen the PIKM in the maxilla and mandible compared to baseline measurements.*

**Null Hypothesis 2:**  *There is no significant difference in PIKM-W augmentation using the present technique and material between the maxilla and mandible.*

## 2. Materials and Methods

### 2.1. Study Design

This prospective case series study examined the clinical outcomes of 24 patients who presented insufficient (<2 mm) width of PIKM in the maxilla and the mandible. Subjects were selected and treated at the Department of Periodontology, Semmelweis University, Budapest, Hungary, between 2018 and 2020. Participants signed a written informed consent statement prior to treatment. The local ethics committee, Semmelweis University Regional and Institutional Committee of Science and Research Ethics, approved the study protocol (approval number: SE RKEB 223/2017). The study was conducted in full accordance with the Declaration of Helsinki, as revised in 2013 [30].

### 2.2. Eligibility Criteria

The inclusion criteria were the following:

- Men and women older than 18 years of age;
- Good compliance to adhere to follow-up protocol and a willingness to commit to a long-term maintenance program after treatment;
- Good oral hygiene (full mouth plaque score, FMPS < 20%);
- PIKM-W either absent or less than 2 mm, measured with a UNC 15 periodontal probe;
- Planned implant placement in the area of keratinised soft-tissue augmentation.

The major exclusion criteria were the following:

- Contributing medical history in which any elective oral surgical intervention would be contraindicated;
- Smoking;
- Pregnant or lactating women;
- Presence of uncontrolled or untreated periodontal disease (full mouth bleeding score > 20%).

### 2.3. Presurgical Treatment

The patients underwent professional oral hygiene instruction and full-mouth supragingival scaling, and 2–400 mg ibuprofen was given as NSAID.

### 2.4. Surgical Treatment

Following local anaesthesia, a horizontal incision was performed at the mucogingival junction (MGJ) using a 15C blade, paying attention not to cut through the periosteum. At the mesial and distal aspects of the initial incision, two vertical releasing incisions were carried out in the mobile mucosa. A split-thickness flap was then gently reflected by the blade or periosteal elevator to prepare a recipient periosteal bed for the graft material based on individual anatomical need. The dimension of the recipient bed was designed to correspond to the planned implant site.

**Figure 1:**
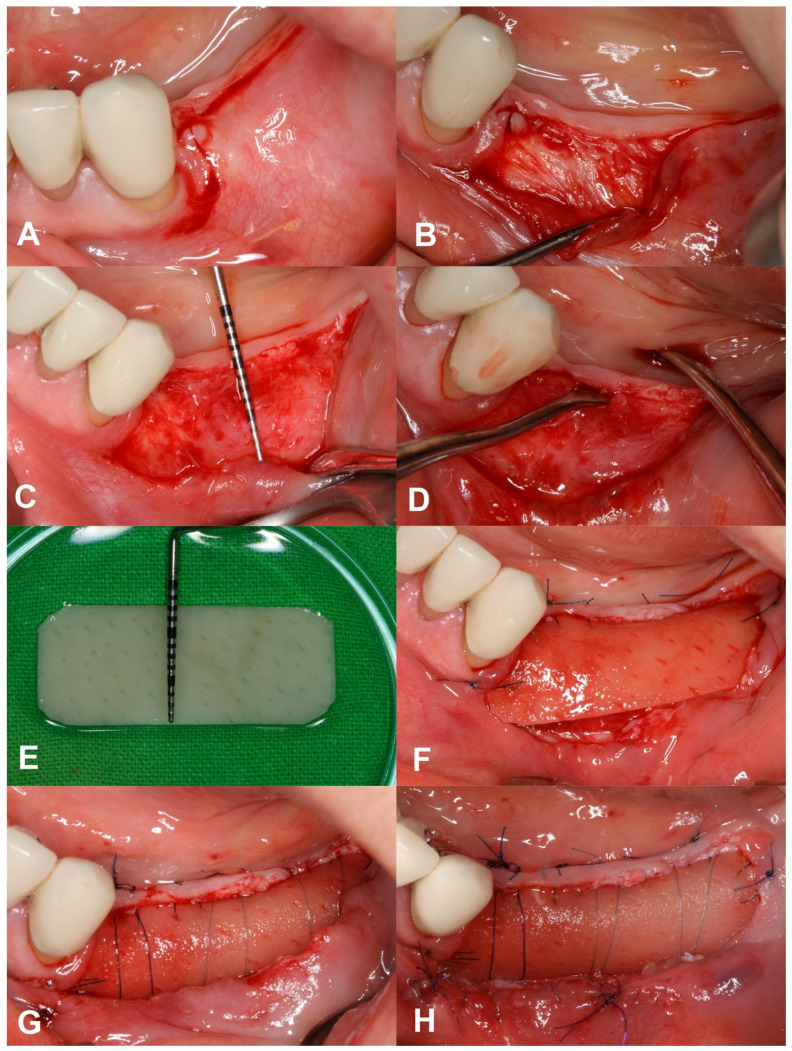
Detailed description of the surgical procedure. (**A**) Split-thickness incision at MGJ. (**B**) Split-thickness mobilisation using a blade or periosteal elevator. (**C**) Recipient site with depth of 10 mm. (**D**) Slight liberation of lingual flap edge to allow the XDM to tuck underneath. (**E**) Trimming and rehydration of the XDM with sterile saline. (**F**) Adaptation and positioning of the XDM towards the attached mucosa using modified mattress and single interrupted sutures. (**G**) Provision of tight contact between periosteum and graft using deep periosteal internal horizontal mattress sutures. (**H**) Immobilisation of the coronal flange of the buccal mucosal flap to the apical part of the same flap using simple continuous suture.

The XDM (mucoderm^®^, Botiss, Zossen, Germany) was trimmed according to the size of the prepared recipient bed (mean width: 9.75 ± 0.62 mm and 8.58 ± 1.16 mm in the maxilla and mandible, respectively) and fully rehydrated with a sterile saline solution. At the coronal part of the recipient bed, the rim of the keratinised mucosa was slightly elevated in order to insert the coronal edge of the XDM underneath. The mesio, mid, and distal coronal parts of the graft were then fixed to/under the attached mucosa via internal (mucosa)/external (graft) horizontal mattress suturing using 5-0 non-resorbable monofilament sutures (Dafilon^®^, B. Braun, Rubí, Spain). After that, deep periosteal internal horizontal mattress sutures were placed above the graft material to compress the XDM to the periosteal bed, making tight contact and avoiding blood clot formation between the recipient site and the XDM. The rim of the mucosal flap was sutured to the apical portion of the same flap via a simple continuous suturing technique using a 6-0 monofilament absorbable suture (Monolac, Chirmax, Prague, Czech Republic), thereby deepening the oral vestibulum as well (Figure 1). The sutures were removed after 14 days. Surgeries were carried out by a periodontal specialist (A.H.).

### 2.5. Postsurgical Instructions and Infection Control

The patients were instructed to avoid mechanical cleaning in the surgical area for two weeks. Instead, chemical plaque control was prescribed with 0.12% chlorhexidine + 0.05% Cetylpyridinium chloride mouth rinse (Paroex, GUM Sunstar, Etoy, Switzerland). The patients were prescribed 250 mg of amoxicillin + clavulanic acid 125 mg (Augmentin 375, GlaxoSmithKline, Brentford, UK) thrice daily for one week. Per individual need, postoperative pain was controlled with diclofenac 50 mg (Cataflam 50, Novartis, Basel, Switzerland). 

The patients were recalled 1 and 2 weeks and 6 and 12 months after surgery for suture removal, plaque control and healing evaluation. Implants were inserted between the 6- and 12-month visits according to individual needs.

### 2.6. Outcome Variables

The primary outcome of the study was to evaluate the overall change in the buccal PIKM-W between baseline and the 6- and 12-month follow-up. PIKM-W was measured as the distance in mm between the mid-crestal line of the edentulous ridge or the mid-buccal margin of the implant and the MGJ using a UNC 15 periodontal probe, rounded to the nearest mm, at baseline and at 6 and 12 months postoperatively. In addition, we compared the possible outcome differences between the maxilla and the mandible. The secondary outcome was to evaluate the dimension of remodelling (shrinkage, contraction) of the XDM using the following formula: (1 − ((PIKM-W at 6 M in mm − PIKM-W baseline in mm)/trimmed XDM width in mm)) × 100 = …%. Measurements were performed by a single calibrated examiner periodontist (X.L.). 

### 2.7. Statistical Analysis 

We performed a patient-based analysis. In the case of multiple sites per patient, one mean value was calculated and utilised in the research. Descriptive statistics were calculated (mean, range and standard deviation) for each study variable. The statistical significance between each group was calculated using Student’s *t*-test (*p*-value < 0.05). The mean values of the PIKM-W were calculated at each examination time point to facilitate intragroup comparison using a paired *t*-test. An intergroup analysis was conducted using an unpaired *t*-test to compare the upper and lower jaw. The statistical analysis was performed in SPSS Statistics V26 (IBM, Armonk, NY, USA).

## 3. Results

### 3.1. Patient Demographics

A total of 24 patients (8 men and 16 women with a mean age of 56.88 ± 12.29 years; range: 33–75 years) were enrolled, with equal distribution in the maxilla and the mandible (Table 1).

### 3.2. Primary Outcome: Changes in PIKM-W

**Figure 2:**
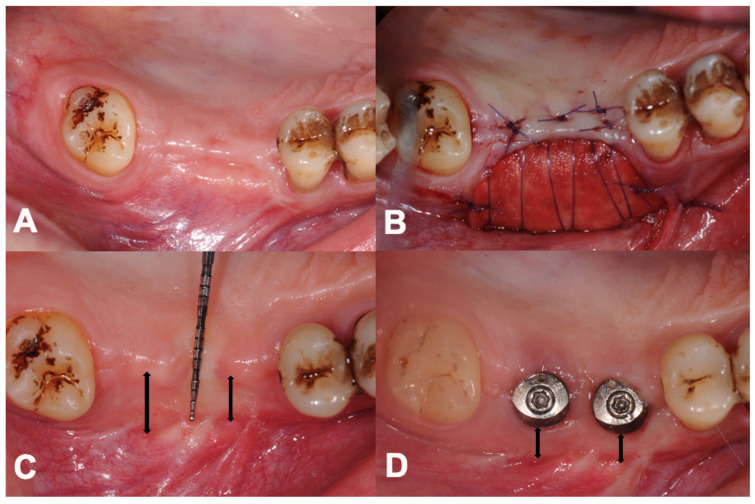
Overview of treatment stages in the upper jaw. (**A**) Lack of sufficient keratinised mucosa preoperatively. (**B**) Immediate view after surgery. (**C**) Six-month follow-up. (**D**) Twelve-month follow-up. (Black two-way arrows indicate the measured buccal width dimensions of the newly formed PIKM).

Intragroup measurements of the change in the PIKM-W were performed separately in both the maxilla and the mandible (Table 2 and Table 3). The mean PIKM-W changed from 0.42 ± 0.47 mm to 3.17 ± 1.21 mm (2.75 ± 0.53 mm difference, *p* < 0.001) at the 6-month follow-up and to 2.36 ± 1.34 mm (1.94 ± 0.62 mm difference, *p* < 0.001) at the 12-month follow-up in the 12 maxillary sites (Figure 2). 

**Figure 3:**
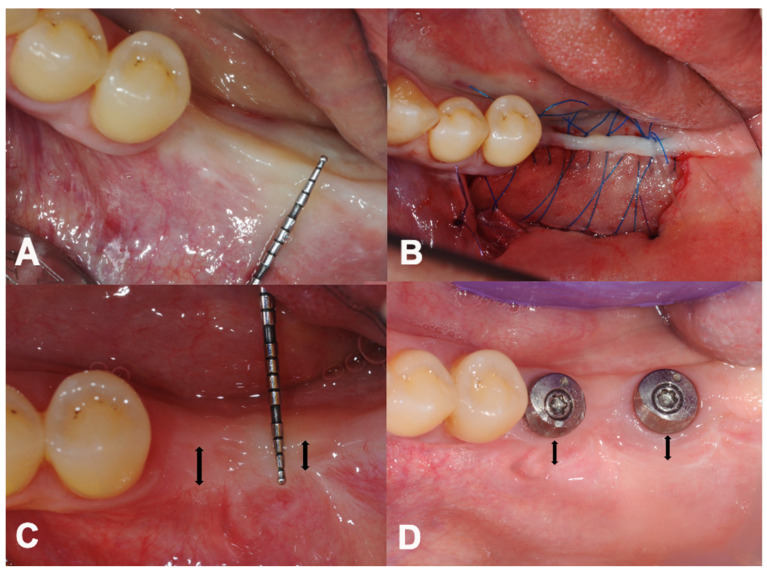
Overview of treatment stages in the lower jaw. (**A**) Lack of sufficient keratinised mucosa preoperatively. (**B**) Immediate view after surgery. (**C**) Six-month follow-up. (**D**) Twelve-month follow-up. (Black two-way arrows indicate the measured buccal width dimensions of the newly formed PIKM).

**Figure 4:**
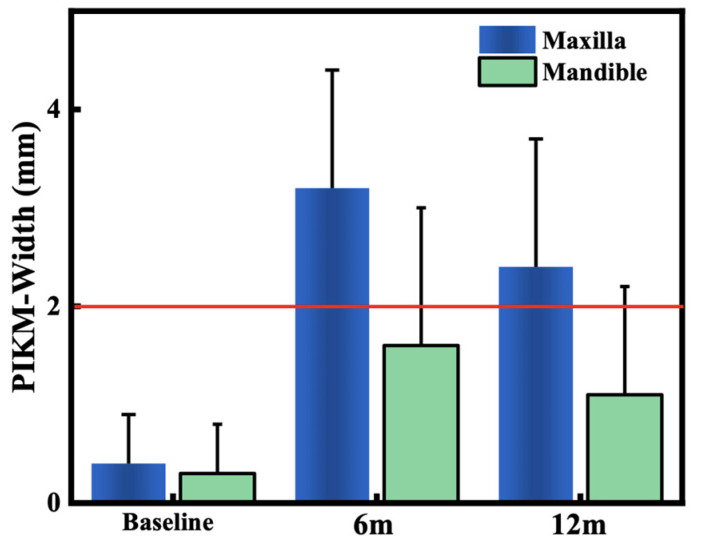
PIKM-W changes in the maxilla and mandible. The red line indicates the 2 mm minimum necessary PIKM-W.

In the 12 mandibular sites (Figure 3), the mean PIKM-W change detected was from 0.29 ± 0.45 mm to 1.58 ± 1.44 mm (1.29 ± 0.70 mm, difference, *p* < 0.001) and 1.08 ± 1.07 (0.79 ± 0.44 mm, difference, *p* = 0.02) at 6 and 12 months, respectively (Figure 4).

### 3.3. Secondary Outcome: Dimension of Graft Remodelling

The decrease in the width of the graft at the 6-month evaluation equalled 67.7 ± 11.8% in the maxilla and 81.6 ± 16.6% in the mandible, and this continued to 75.9 ± 13.9% and 87.4 ± 12.3% at 12 months in the maxilla and mandible, respectively. A statistically significant intergroup difference was detected between the maxilla and mandible at 6 as well as 12 months (*p* < 0.05).

## 4. Discussion

The present study demonstrated a safe and uneventful postoperative period of the investigated technique and material for increasing PIKM-W. In addition, despite the marked graft remodelling, a statistically significant and clinically sufficient (>2 mm) amount of new keratinised mucosa was achieved in the maxilla. Although the widening of the PIKM was also significant in the mandibular sites, the desired minimum width was reached with varying success. 

In order to reconstruct the desirable minimum amount of 2 mm of PIKM-W, ECTG (FGG) is widely regarded as the most effective method due to its ability to minimise soft-tissue contraction after surgery and provide the highest level of predictability [13,16,17,18,19,21]. A study by Schmitt et al. reported that ECTG-augmented PIKM-W remained 8.4 mm after a 5-year follow-up [31]. The systematic review by Thoma et al. reported that an apically positioned flap (APF) combined with ECTG resulted in an excellent improvement in peri-implant health [1]. A similar conclusion was drawn by Vignoletti et al., who found that the mean PIKM-W increased by 4.5 mm using the augmentation procedure of APF plus autograft [32]. Nevertheless, harvesting the ECTG has the potential to induce substantial pain and excessive bleeding, and it compromised healing at the donor sites. In addition, it is essential to note that there is a restricted supply of tissue, and achieving a precise colour match at the recipient location is suboptimal, as indicated by previous studies [17,18,22,23].

In order to address the constraints of ECTG, researchers have proposed using a xenogeneic collagen matrix (XCM) to enhance the potential growth in peri-implant keratinised mucosa width (PIKM-W). Sanz et al. reported a mean increase of 2.5 mm in the keratinised mucosa width using XCM (Mucograft^®^, Geistlich Pharma AG, Wolhusen, Switzerland) [24,26,27]. Their study documented a shrinkage rate of 67% [24]. Another study, by Lorenzo et al., reported that PIKM-W augmentation procedures using XCM (Mucograft^®^) and connective tissue graft achieved a mean value of 2.75 mm and 2.8 mm, respectively [25]. However, both investigations were discontinued at 6 months. The literature indicates that XCM has yielded favourable outcomes in augmenting the width of keratinised mucosa. Nevertheless, XCM is subjected to swift resorption/remodelling due to its porous surface and loose structure. This leads to a significantly lower amount of keratinised tissue during open healing than is the case with ECTG [31].

A novel xenogeneic dermal matrix of porcine origin composed of I/III type collagen (XDM, mucoderm^®^) has been recently introduced as a potential substitution for autograft in periodontology and implant dentistry.

According to a study by Pabst et al., this material showed excellent tissue integration and rapid revascularisation [33]. Park et al. explored the cell proliferation characteristics of mucoderm^®^. Host cell migration and penetration in this matrix were complemented by its interconnected structure, which allowed microvessel formation and neo-angiogenesis [34]. Some studies have investigated the immune response of this XDM and revealed that its natural 3D matrix scaffold structure prolongs the degradation period and improves its mechanical properties, which is an additional advantage when slow resorption and keratinisation are targets during “open”, secondary wound healing [32,35].

In a recent retrospective investigation, Zafiropoulos et al. utilised XDM to increase the PIKM-W, and a 5.4 mm mean growth was reported after the 6-month follow-up [28]. Two-thirds of the sites were located in the maxilla. Nevertheless, the same amount of shrinkage was reported in both arches at all site locations (3.9 ± 1.2 mm vs. 3.9 ± 1.1 mm and 3.9 ± 1.3 mm vs. 3.9 ± 1.1 mm in the maxilla vs. mandible and anterior vs. posterior sites, respectively).

Our data revealed a smaller decrease in graft width in the maxilla (67.7 ± 11.8%) in contrast to the mandible (81.6 ± 16.6%) and also revealed statistically significant differences between the two jaws in PIKM-W change and in the magnitude of graft remodelling at 6- (*p* = 0.008; *p* = 0.03) and 12-month (*p* = 0.02; *p* = 0.04) intervals. The reason for these results may originate in the anatomic differences, since the mandibular sites present with a shallower vestibule and more coronal adhesion of the more voluminous buccal muscles. Therefore, instead of graft contraction or shrinkage, this may be more likely defined as the coronal migration of the MGJ as a result of undesired but natural soft-tissue remodelling. Nandkeoliar et al. demonstrated that this interarch divergence is around 2 mm in the age group of our subjects (11.28 ± 2.31 in the maxilla and 9.42 ± 2.87 in the mandible) [36]. Based on these differences, the maxillary and mandibular regions should be investigated separately. The cumulative PIKM-W outcomes of both arches in our investigation would have led to different results and conclusions than the separate ones. This should be considered in further study designs; otherwise, it may lead to deceptive outcomes that could potentially impact clinical decision making. 

The other strength of the present trial is the 1-year length of the investigation period. Our results indicate that the newly formed PIKM remodelling continues from 6 to 12 months, reaching statistical significance (*p* = 0.009 maxilla; *p* = 0.03 mandible). Therefore, a minimum investigation period of 12 months should be considered when seeking long-term tissue stability. 

According to the authors’ knowledge, this prospective case series investigation is the only study to test this novel XDM in peri-implant soft-tissue augmentation in the maxilla and mandible, with 12 months of follow-up. When comparing the results of this study to other investigations, it needs to be highlighted that not only the material but also the surgical technique could play a crucial role. Based on our previous pilot aiming at fast revascularisation and prevention of graft detachment, it is essential to attain tight contact between the XDM and the periosteal bed. A commonly used surgical technique anchors the graft to the adjacent flaps with single interrupted sutures. Due to a lack of immobility and insufficient connection between the graft and the periosteum, this technique may create blood clot formation in between and, thereby, cause graft disintegration. This, and the insufficient apical locking of the mobile mucosa, may lead to considerable remodelling of the graft and coronal remigration of the mobile non-keratinised mucosa. 

The newly formed tissue should not only be keratinised but also attached to the mucosa. To reach this goal, we applied modified internal/external horizontal deep periosteal mattress sutures to stabilise the graft to the periosteum and fixed epithelium only and avoided apical/lateral sutures to the mobile mucosa. Furthermore, the coronal edges of the graft were inserted under the keratinised epithelium. In order to keep the split-thickness mucosal flap edge as apical as possible, the coronal rim of the flap was folded to the most apical part of the same flap in such a way that its non-keratinised epithelium adhered to the periosteum and fixed with simple continuous sutures. We aimed to disclose a step-by-step description of our surgical procedure in this manuscript.

The slightly different measurement landmark at 12 M could be considered a limitation of the study. The reason for this was the “put patients’ benefit first” research initiative, as we believed it would not have been ethically correct to expect patients to wait a whole year to receive their fixtures, when the sites were ready to be implanted. In addition, implants were placed following midcrestal incision; thereby, the PIKM dimensions were not altered but only buccally shifted.

While the study achieved statistical significance in several outcomes, the clinical significance of these findings, particularly in terms of long-term implant survival and patient-centred outcomes, remains to be established. Future studies should focus not only on the statistical analysis of clinical parameters but also on the impact of these changes on implant success rate, patients’ quality of life, oral function, brushing discomfort, aesthetic outcomes, etc.

Due to the continued remodelling after 6 months, 12-month investigations are warranted. In order to draw broader conclusions and provide more predictable solutions in the lower jaw, randomised controlled clinical trials with large sample sizes and with an ECTG control group as gold standard are needed in this field.

## 5. Conclusions

- The investigated surgical technique using XDM was safe and significantly widened the PIKM in both jaws.
- The new PIKM formation surpassed the clinically desired 2 mm width in the maxilla. The mandibular sites presented with more variable success.
- The remodelling of the newly formed PIKM continued after 6 months.
- The investigated XDM alone could be considered as a suitable alternative to autograft to reconstruct an adequate amount of keratinised mucosa width in the maxilla.

With respect to the nature of a case series, the conclusions of the present study have to be interpreted carefully.

## Figures and Tables

**Table 1:** Demographic data and site distribution.

	Maxilla	Mandible
Age	55.92 ± 14.38	57.71 ± 11.2
Male	4	0
Female	8	12
Premolar	3	4
Molar	9	8

**Table 2:** Changes in PIKM-W in the maxilla.

Time	Mean	SD	*p*-Value
Initial	0.42	0.47	0.00002
6 months	3.17	1.21	
Difference	2.36	1.34	
Initial	0.42	0.47	0.00035
12 months	2.36	1.34	
Difference	1.94	1.37	
6 months	3.17	1.21	0.009
12 months	2.36	1.34	
Difference	0.81	0.09	

**Table 3:** Changes in PIKM-W in the mandible.

Time	Mean	SD	*p*-Value
Initial	0.29	0.45	0.010
6 months	1.58	1.44	
Difference	1.29	0.70	
Initial	0.29	0.45	0.023
12 months	1.08	1.07	
Difference	0.79	0.44	
6 months	1.58	1.44	0.033
12 months	1.08	1.07	
Difference	0.50	0.27	

## Data Availability

Data are contained within the article.
